# AdipoRon prevents myostatin‐induced upregulation of fatty acid synthesis and downregulation of insulin activity in a mouse hepatocyte line

**DOI:** 10.14814/phy2.14152

**Published:** 2019-06-27

**Authors:** Xin‐Hua Liu, Jiang Ping Pan, William A. Bauman, Christopher P. Cardozo

**Affiliations:** ^1^ National Center for the Medical Consequences of Spinal Cord Injury James J. Peter VA Medical Center Bronx New York; ^2^ Department of Medicine Icahn School of Medicine at Mount Sinai New York New York; ^3^ Department of Rehabilitation Medicine Icahn School of Medicine at Mount Sinai New York New York

**Keywords:** AdipoRon, fatty acid metabolism, hepatocytes, myostatin

## Abstract

Liver diseases such as non‐alcoholic fatty liver disease (NAFLD) and non‐alcoholic steatohepatitis (NASH) are characterized by excess hepatic accumulation of lipid droplets and triglycerides which are associated with defective insulin action. Myostatin (Mstn) and adiponectin, secreted by muscle cells and adipocytes, respectively, play important roles in regulating insulin signaling and energy metabolism. The mechanisms underlying the actions of Mstn and adiponectin remain largely unknown. Moreover, the interactions between Mstn and adiponectin in regulating gene expression critical for fatty acid metabolism and insulin action in hepatocytes have not been investigated. The effects of Mstn and AdipoRon, a synthetic adiponectin receptor agonist that is orally active, alone or in combination, on hepatic gene expression and function was investigated. While Mstn increased fatty acid (FA) accumulation and desensitized cellular responses to insulin, AdipoRon protected against Mstn‐induced defects in hepatic gene expression and function. In addition, these effects of Mstn were associated with reduced AMPK and PPAR *α* activities which were reversed by AdipoRon. Finally, AdipoRon was able to prevent Mstn‐induced activation of the Smad2/3 pathway. These data suggest crosstalk between Mstn‐induced Smad2/3 and adiponectin‐induced AMPK/PPAR *α* pathways, which may play important roles in the regulation of hepatic gene expression critical for FA metabolism and insulin signaling. In addition, the data suggest that AdipoRon, as an adiponectin receptor agonist, may serve a therapeutic role to reduce the hepatic contribution to the disorders of fat metabolism and insulin action.

## Introduction

Hepatocytes are parenchymal cells of the liver that are responsible for packaging dietary or de‐novo synthesized lipid for release into the circulation and storing excess lipids in the form of lipid droplets, thus making the liver the primary organ responsible for lipid homeostasis (Huang et al. 2011). The liver is susceptible to various insults resulting in pathologies such as non‐alcoholic fatty liver disease (NAFLD) and non‐alcoholic steatohepatitis (NASH), both characterized by excess hepatic accumulation of lipid droplets and triglycerides which may develop as a consequence of alterations in *β*‐oxidation and fatty acid synthesis pathways (Huang et al. 2011). NAFLD and NASH are common complications of obesity and strong risk factors for insulin resistance and metabolic syndrome (Duseja et al. 2015; Maratos‐Flier 2017). However, there is still controversy about dozens of factors that have been implicated as potential mediators. It is also not clear how hepatic lipid droplets accumulate in the liver, or what molecular events regulate the interaction among elevated fatty acid levels, insulin resistance, and metabolic syndrome (Maratos‐Flier 2017).

Myostatin (Mstn), a member of the transforming growth factor‐*β* (TGF‐*β*) superfamily, is a critical autocrine/paracrine inhibitor of skeletal muscle growth and development (McPherron et al. 1997). Beyond the confines of its traditional role, Mstn has recently been shown to play an important role in metabolism (Lin et al. 2002; Guo et al. 2009; Deng et al. 2017). In animal studies, genetic knockdown of Mstn increased muscle mass, reduced fat mass, and protected against dietary‐induced insulin resistance (McPherron and Lee 2002; Zhao et al. 2005). In addition, Guo et al. 2009 showed that Mstn knockout‐induced muscle gain and fat loss are associated with elevated glucose utilization and insulin sensitivity. Moreover, elevated levels of Mstn in muscle and plasma have been associated with obesity, and type‐1 and type‐2 diabetes in both human and mouse models (Milan et al. 2004; Chen et al. 2009; Hittel et al. 2009, 2010). While a negative regulatory effect of Mstn on metabolism is widely reported, the literature contains some contradictory findings (Zimmers et al. 2002; Mitchell et al. 2006; Zhang et al. 2011a, 2011b; Rodgers 2014). Mstn is secreted from muscle and thus can act locally or systemically via activin type II B (ActRIIB) receptors (Thomas et al.^2000^; Rebbapragada et al. 2003; Ricaud et al. 2003; Zhu et al. 2004; Han et al. 2013), by which it facilitates biological crosstalk between organ systems and regulates metabolic processes (Tsuchida 2004; Zhang et al. 2011a, 2011b; Fennen et al. 2016). Increased circulating Mstn levels are observed in patients with liver disease (Garcia et al. 2010) and are associated with worse survival in patients with liver cirrhosis (Nishikawa et al. 2017). Moreover, Mstn has been shown to inhibit liver cell proliferation and glucose uptake, as well as alter hepatic gene expression critical for lipogenic and insulin signaling in both in vitro and in vivo mouse models (Wilkes et al. 2009; Guo et al. 2013; Watts et al. 2014). These studies suggest that the liver is an important target for Mstn action and that Mstn contributes to altered lipid homeostasis and diminished regenerative capacity in liver diseases. Despite these intriguing findings, the details of the roles for Mstn in liver lipid homeostasis and insulin action remain poorly delineated.

Adiponectin, secreted by adipocytes, is the most abundant adipokine in the serum and modulates lipid and glucose metabolism. Adiponectin binds to two specific receptors, AdipoR1 and AdipoR2, which are differentially expressed among tissues. AdipoR1 is abundantly expressed in skeletal muscle, whereas AdipoR2 is predominantly expressed in the liver (Yamauchi et al. 2003). In the liver, adiponectin regulates both glucose and lipid homeostasis and has an insulin‐sensitizing effect (Gamberi et al. 2018). The binding of adiponectin to its specific receptors triggers the activation of a signaling cascade that becomes altered in liver physiology and pathologies. The activation of AdipoR1 promotes AMP‐activated kinase (AMPK) activity while activated AdipoR2 stimulates peroxisome proliferator‐activated receptor *α* (PPAR*α*) signaling (Yamauchi et al. 2003). AMPK activation inhibits gluconeogenesis and suppresses lipid synthesis via inhibitory phosphorylation of acetyl‐CoA carboxylase (ACC), a rate‐limit enzyme that controls fatty acid synthesis, leading to promotion of lipid catabolism and preventing TG formation in the liver (Misra 2008). On the other hand, PPAR*α* activation is thought to be involved in adiponectin‐stimulated fatty acid ß‐oxidation, but not glucose uptake (Yamauchi et al. 2003; Kersten and Stienstra 2017). Animal studies have demonstrated that adiponectin possesses potent protective activities against various forms of liver disease (Wang et al. 2008) and displays regenerative properties for the liver (Ezaki et al. 2009). Tomita et al. reported that enhanced expression of AdipoR2 in the liver improves NASH at every stage, from the early stage to the progression of fibrosis, whereas inhibition of AdipoR2 diminishes hepatic PPAR*α* activity leading to an increase in lipid peroxidation (Tomita et al. 2008). Moreover, several clinical and animal studies have revealed a negative correlation between adiponectin levels and several liver diseases, including NAFLD, NASH, hepatic fibrosis and hepatocellular carcinoma. These findings suggest that hypoadiponectinemia in obese people may be an important risk factor for the clinical progression of these chronic live diseases (Gamberi et al. 2018).

Several lines of evidence have recently established that both skeletal muscle and adipose tissue are endocrine organs that produce and release soluble mediators referred to as myokines and adipokines, respectively, which act in a paracrine or endocrine fashion to modulate metabolism by signaling to other organs (Pedersen and Febbraio 2012; Gamberi et al. 2018). Mstn has been linked to liver diseases as a potential negative regulator (Guo et al. 2013; Nishikawa et al. 2017), whereas adiponectin has been demonstrated to have a beneficial role in the liver (Gamberi et al. 2018). The crosstalk between Mstn and adiponectin, as well as interactive effects that modulate the molecular events in the metabolic homeostasis process in the liver have not been elucidated. AdipoRon, an orally active synthetic small‐molecule agonist of adiponectin receptors, has been shown to exert effects similar to those of adiponectin in both muscle and liver. AdipoRon is able to activate both AdipoR1 and R2 (Okada‐Iwabu et al. 2013) causing elevated activity of AMPK and PPAR*α* pathways, and ameliorates insulin resistance and glucose intolerance in mice fed a high‐fat diet (Zhang et al. 2015). These data imply that AdipoRon could be the basis for a novel therapeutic approach for obesity related diseases. However, whether AdipoRon possess hepatoprotective properties, in particular, against Mstn‐induced liver cell lesions and dysregulation of hepatic gene expression, has not been investigated. To address this possibility, the effects of Mstn and AdipoRon, alone or in combination, on hepatic gene expression critical for lipid metabolism and insulin action, and to examine the crosstalk between Mstn‐induced Smad2/3 and AdipoRon‐induced adipoR1 & R1 pathways in a mouse hepatocyte line, FL83B were investigated.

## Materials and Methods

### Cell culture and reagents

FL83B, a hepatocyte cell line derived from a normal liver taken from 15 to 17 day old fetal mice, was purchased from ATCC (Manassas, VA). Cells were maintained in F‐12K medium containing 10% FBS supplemented with 1% penicillin/streptomycin at 37°C. Before treatments with various compounds, cells were washed with PBS, and the medium containing 2% FBS was replaced overnight. The purpose of using 2% FBS medium for the cell treatment was to reduce basal cellular activity and bring all cells to the phase of growth arrest thereby equalizing all cells into the same phase of cell cycle which enables pronounced effect following treatment with growth signals (Zetterberg and Larsson 1985; Van Rechem et al. 2010). It also provides more reproducible experimental conditions (Colzani et al. 2009). Recombinant mouse myostatin protein was obtained from R&D Systems (Minneapolis, MN), AdipoRon was purchased from Sigma‐Aldrich (St Louis, MO), human recombinant insulin is a product from MP Biomedicals (Santa Ana, CA).

### Preparation of cell lysates and Western blotting (WB)

Cells were rinsed twice with ice‐cold PBS and scraped off the plates with 1.5 mL of PBS containing 4 mmol/L iodoacetate. After centrifugation, the pellets were resuspended in CHAPS extraction solution (10 mmol/L CHAPS, 2 mmol/L EDTA, pH 8.0, and 4 mmol/L iodoacetate in PBS) with protease inhibitors. The samples were incubated for 30 min on ice and centrifuged at 15,000*g* for 10 min. The supernatants were collected and stored at −70°C. Proteins from the nuclear fractions were isolated using a commercial kit from Pierce (Rockford, IL), according to the manufacturer's instructions. For immunoblotting, cell lysates were electrophoresed on SDS‐polyacrylamide gels, electrophoretically transferred to a PVDF membrane, and incubated with primary antibodies overnight at 4°C. *β*‐tubulin and PCNA antibodies were used as loading controls for total cell lysates and nuclear protein, respectively. Antibodies against ACC/phospho‐ACC(Ser79), AMPK/phospho‐AMPK (T172), AKT/phospho‐AKT (Ser473; Thr308), IRS1/phospho‐IRS1 (Tyr895), IRS‐2, Smad2/3/phospho‐Smad2/3, FAS, Perillipin‐2 and PCNA were purchased from Cell Signaling (Danvers, MA). Antibodies against PPAR*α*, AdipoR1, and *β*‐tubulin were products of Abcam (Cambridge, MA). AdipoR2 was obtained from Thermo‐Fisher (Waltham, MA).

### Quantitative real‐time (Rt) PCR

Total RNA was exacted using RNasey mini kits (QIAGEN). 1 *μ*g of total RNA was used to synthesize cDNA using High Capacity RNA‐to‐cDNA kit (Applied Biosystems). Relative expression of mRNA was determined by *q*Rt‐PCR, as described previously (Liu et al. 2013) using a thermocycler (model ViiA7, Applied Biosystems). Specific primers and *Tag*Man Universal Master Mixer were purchased from Applied Biosystems. *Tag*Man Reaction Mixes contained 3 *μ*L cDNA (10 ng/*μ*L). For each sample, the determinations were performed in triplicate, and the means for the crossing points of triplicates were used in subsequent calculations. Relative mRNA levels were expressed as fold‐change using the 2^−ΔΔCt^ method. Data were normalized relative to 18s RNA.

### PPARα transcription factor assay and preparation of nuclear extracts

The binding capacity of PPAR*α*, specifically to the PPRE, was assayed using a non‐radioactive, sensitive method for detecting specific transcription factor DNA binding activity in nuclear extracts. This assay replaces the cumbersome radioactive electrophoretic mobility shift assay (EMSA). The assay kit was purchased from Abcam according to the manufacturer's instructions. Nuclear extracts were isolated using a commercial kit from Pierce (Rockford, IL), according to the manufacturer's instructions.

### Triglyceride content assay

TG content in cell lysates extracted from treated cells was assayed using an assay kit from Cayman Chemical Inc. (Ann Arbor, MI).

### Statistics

Data are expressed as means ± SEM. The significance of differences between pairs of means was determined with an unpaired two‐tailed Student's *t*‐test. Statistical calculations were performed with Prism 6.0 (GraphPad).

## Results

### AdipoRon prevented myostatin‐induced fatty acid accumulation

**Figure 1:**
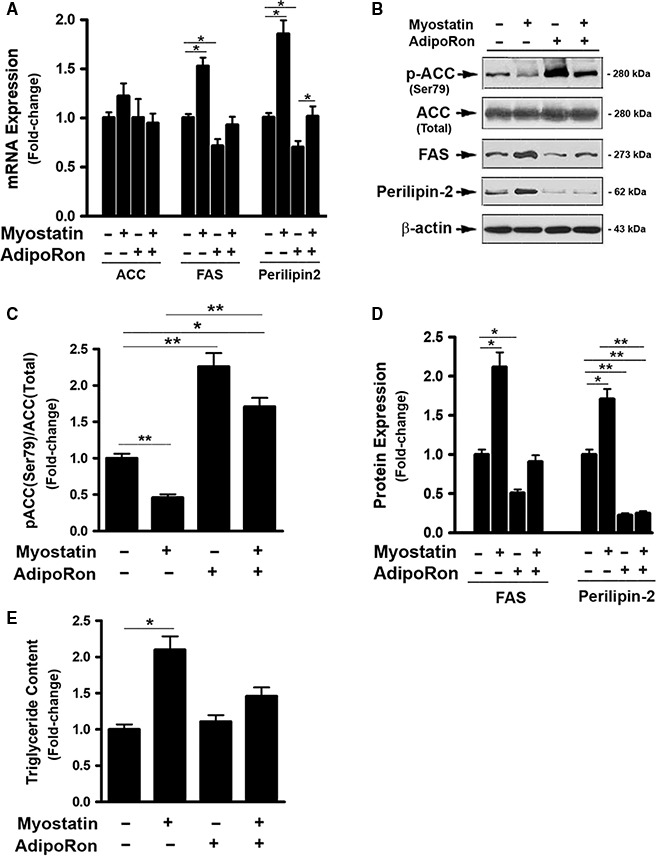
AdipoRon prevented myostatin‐induced fatty acid accumulation. (A) FL83B cells were treated with either vehicle or myostatin (100 ng/mL), or AdipoRon (20 *μ*mol/L) for 2d, total RNA was isolated and subjected to Rt‐PCR analysis. (B) Cells treated with either vehicle or myostatin (100 ng/mL), or AdipoRon (20 *μ*mol/L) for 3d. Total cell lysates were prepared and subjected to western blotting. (C) Blots in B (2‐top panels) were quantified by scanning densitometry and the ratio of phosphor‐ACC (Ser79) to endogenous ACC was calculated and is shown as fold of change. (D) Blots in B (3‐bottom panes) were quantified by scanning densitometry and the ratio of FAS or perilipin2 to *β*‐actin was calculated and is shown as fold of change. (E) Cells treated with either vehicle or myostatin (100 ng/mL), or AdipoRon (20 *μ*mol/L) for 3d. Total cell lysates were prepared and subjected to triglyceride content assay. Data shown in B are representative Western blot analysis; Data shown in A, C, D, E, are mean values ± SEM from three separate determinations; *P < 0.05; **P < 0.01 compared to vehicle‐treated cells.

To determine the interactive role of Mstn and AdipoRon in fatty acid (FA) metabolism in the liver, the effects of Mstn and AdipoRon, alone or in combination, on the expression and activation of hepatic genes critical for FA metabolism were determined. Mstn treatment had no significant effect on mRNA and protein expression of acetyl‐coenzyme A carboxylase (ACC), a rate‐limiting enzyme controlling FA synthesis (Fig. 1A and B). However, treatment of FL83B cells with Mstn for 3d activated ACC by significantly inhibiting phosphorylation of Ser79 (Fig. 1B and C), a typical inhibitory site of ACC activity, compared to vehicle‐treated cells. In addition, cells treated with Mstn demonstrated increased mRNA expression for fatty acid synthase (FAS) and perilipin (PLIN)2 (Fig. 1A), a lipid droplet binding protein involved in the storage of neutral lipid within lipid droplets. Western blotting also revealed upregulation of FAS and PLIN2 protein levels in Mstn‐treated cells (Fig. 1B and D). In contrast, AdipoRon alone increased inhibitory phosphorylation of ACC at Ser79 (Fig. 1B and C). In addition, AdipoRon‐treatment significantly inhibited the expression of FAS and PINL2 mRNA and protein (Fig. 1A, B and D). When the cells were treated with a combination of Mstn and AdipoRon, ACC phosphorylation was increased above levels in control cells, coincident with downregulation of FAS and PLIN2 mRNA and protein expression compared to control cells. To test the biological consequences of altered hepatic gene expression, intracellular triglyceride (TG) content was examined. AdipoRon treatment alone had no significant effect. However, Mstn treatment for 3d led to an increase in intracellular TG content but this increase in lipid was not observed when the combination of Mstn and AdipoRon treatment was tested (Fig. 1E).

### AdipoRon protected against myostatin‐induced insulin resistance

**Figure 2:**
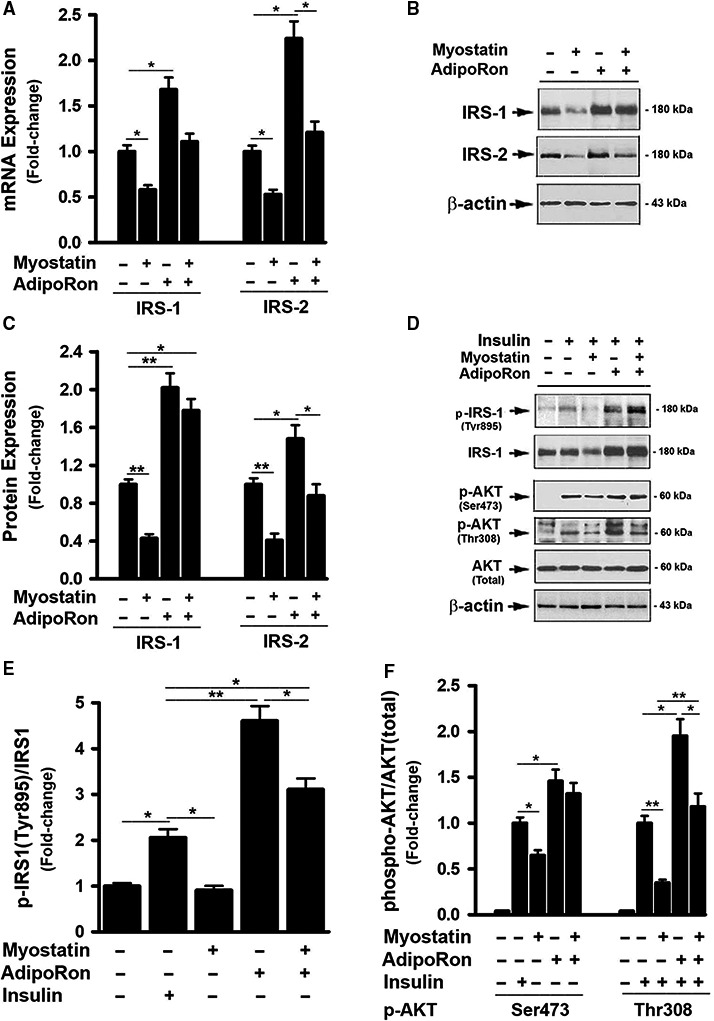
AdipoRon protected against myostatin‐induced insulin resistance. (A) FL83B cells were treated with either vehicle or myostatin (100 ng/mL), or AdipoRon (20 *μ*mol/L) for 2d, total RNA was isolated and subjected to Rt‐PCR analysis. (B) Cells treated with either vehicle or myostatin (100 ng/mL), or AdipoRon (20 *μ*mol/L) for 3d. Total cell lysates were prepared and subjected to western blotting. (C) Blots in B were quantified by scanning densitometry and the ratio of IRS‐1 or IRS‐2 to *β*‐actin was calculated and is shown as fold of change. (D) Cells were pretreated with either vehicle or myostatin (100 ng/mL), or AdipoRon (20 *μ*mol/L) for 3d, followed by the treatment with insulin (100 nmol/L) for 30 min. Total cell lysates were prepared and subjected to western blotting. (E) Blots in D (panels 1 & 2 from top) were quantified by scanning densitometry and the ratio of phosphor‐IRS1 (Tyr895) to endogenous IRS1 was calculated and is shown as fold of change. (F) Blots in D (panels 3, 4, 5 from top) were quantified by scanning densitometry and the ratio of phosphor‐AKT (Ser473) or phosphor‐AKT (Thr308) to endogenous AKT was calculated and is shown as fold of change. Data shown in B and D are representative Western blot analysis; Data shown in A, C, E, F are mean values ± SEM from three separate determinations; *P < 0.05; **P < 0.01 compared to vehicle‐treated cells.

A link between adiponectin, Mstn and insulin has been reported (Liu et al. 2012, 2018; Gamberi et al. 2018). The interactive effect of these two factors on hepatic gene expression critical to insulin signaling was therefore examined. Mstn treatment led to significant inhibition of expression of IRS‐1 and IRS‐2 mRNA and protein (Fig. 2A–C). Cells treated with Mstn for 3d followed by insulin treatment for 20 min showed decreased phosphorylation of IRS‐1 at the site of Tyr895 compared to vehicle‐treated cells, indicating a reduced response of the cells to insulin stimulation (Fig. 2D and E). Mstn also suppressed AKT phosphorylation at both Ser473 and Thr308 sites compared with control cells (Fig. 2D and F). These results implied a role for Mstn in desensitizing the cellular response to insulin. In contrast, treating with AdipoRon alone increased IRS‐1 & IRS‐2 expression, both mRNA and protein levels (Fig. 2A–C), promoted insulin sensitivity evidenced by enhanced IRS‐1 (Tys895) and AKT (Ser473 and The308) phosphorylation (Fig. 2D–F). When cells were treated with a combination of Mstn and AdipoRon, the inhibitory effects of Mstn on IRS‐1 and IRS‐2 gene expression, protein levels, and insulin signaling were abolished (Fig. S2).

### AdipoRon prevented myostatin‐induced inhibition of AdipoR1 activity

**Figure 3:**
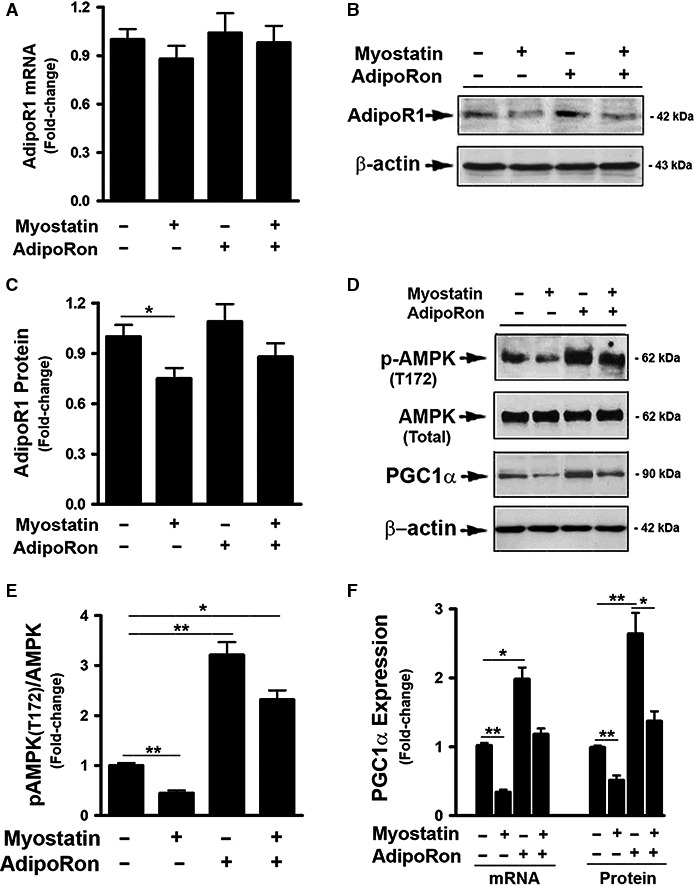
AdipoRon prevented Myostatin‐induced inhibition of AdipoR1 activity. (A) FL83B cells were treated with either vehicle or myostatin (100 ng/mL), or AdipoRon (20 *μ*mol/L) for 2d, total RNA was isolated and subjected to Rt‐PCR analysis. (B, D) Cells were treated with either vehicle or myostatin (100 ng/mL), or AdipoRon (20 *μ*mol/L) for 3d. Total cell lysates were prepared and subjected to western blotting. (C) Blots in B were quantified by scanning densitometry and the ratio of AdipoR1 to *β*‐actin was calculated and is shown as fold of change. (E) Blots in D (panels 1 & 2, from top) were quantified by scanning densitometry and the ratio of phosphor‐AMPK (T172) to endogenous AMPK was calculated and is shown as fold of change. (F) Blots in D (panels 3 & 4, from top) were quantified by scanning densitometry and the ratio of PGC1*α* to *β*‐actin was calculated and is shown as fold of change. Data shown in B and D are representative Western blot analysis; Data shown in A, C, E, F are mean values ± SEM from three separate determinations; *P < 0.05; **P < 0.01 compared to vehicle‐treated cells.

Liver cells express both types of adiponectin receptor, that is, AdipoR1 and AdipoR2 (Yamauchi et al. 2003). AdipoRon is able to activate AdipoR1 leading to an elevated AMPK activity (Okada‐Iwabu et al. 2013). To further understand the interactions between Mstn and AdipoRon on regulating hepatic genes, the effect of Mstn on AdipoR1 was tested in the presence or absence of AdipoRon. In FL83B cells, treatment with Mstn had no effect on AdipoR1 mRNA expression (Fig. 3A), but downregulated AdipoR1 protein levels compared with vehicle‐treated cells (Fig. 3B and C). In addition, Mstn treatment significantly suppressed AMPK activation as evidenced by reduced AMPK (Thr172) phosphorylation (Fig. 3D and C). Moreover, Mstn suppressed the expression of mRNA and protein of PGC1*α* (Fig. 3D and F), a direct down‐stream target of AMPK activation and a key regulator of mitochondrial biogenesis (Jager et al. 2007; Irrcher et al. 2008), supporting the conclusion that signaling downstream of AMPK was inhibited as a result of lower AMPK (T172) phosphorylation. The inhibitory effects of Mstn on AdipoR1 expression and activity were reversed by the addition of AdipoRon (Fig. 3).

### AdipoRon prevented myostatin‐induced inhibition of AdipoR2 activity

**Figure 4:**
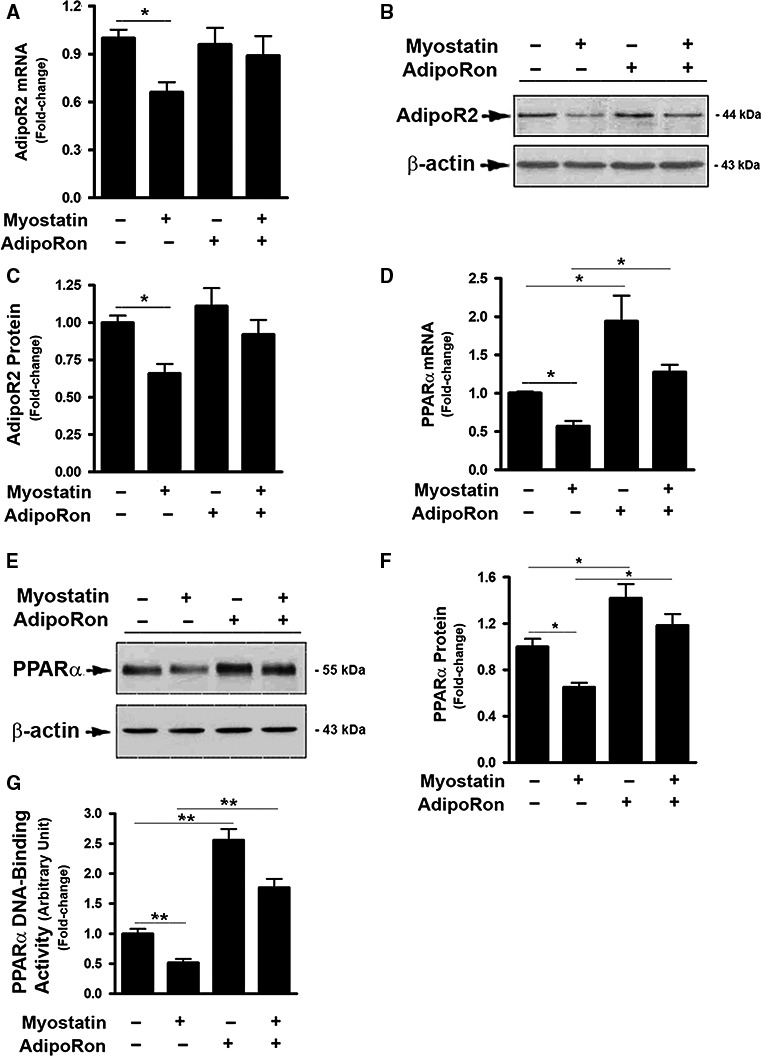
AdipoRon prevented myostatin‐induced inhibition of AdipoR2 activity. (A, D) FL83B cells were treated with either vehicle or myostatin (100 ng/mL), or AdipoRon (20 *μ*mol/L) for 2d, total RNA was isolated and subjected to Rt‐PCR analysis. (B, E) Cells were treated with either vehicle or myostatin (100 ng/mL), or AdipoRon (20 *μ*mol/L) for 3d. Total cell lysates were prepared and subjected to western blotting. Blots in B and E were quantified by scanning densitometry and the ratio of AdipoR2 to *β*‐actin (C) or PPAR *α* to *β*‐actin (F) was calculated and is shown as fold of change. (G) Cells were treated with either vehicle or myostatin (100 ng/mL), or AdipoRon (20 *μ*mol/L) for 3d. Nuclear protein was prepared and subject to DNA‐binding assay. Data shown in B and E are representative Western blot analysis. Data shown in A, C, D, F, G are mean values ± SEM from three separate determinations; *P < 0.05; **P < 0.01 compared to vehicle‐treated cells.

AdipoR2 is predominantly expressed in the liver (Yamauchi et al. 2003). Thus, the effects of Mstn on AdipoR2 were examined. As shown in Figure 4, Mstn‐treated cells demonstrated reduced expression of AdipoR2 mRNA and protein. Mstn also inhibited AdipoR2 activation as demonstrated by decreased levels of PPAR*α* mRNA and protein (Fig. 4D–F), as well as decreased DNA‐binding capacity of PPAR*α* (Fig. 4G). Treatment with AdipoRon alone had no effect on AdipoR2 expression, but upregulated PPAR*α* mRNA and protein levels with a dramatic increase in PPAR*α* DNA‐binding capacity (Fig. 4D–G).

### AdipoRon prevented myostatin‐induced activation of Smad2/3 signaling

**Figure 5:**
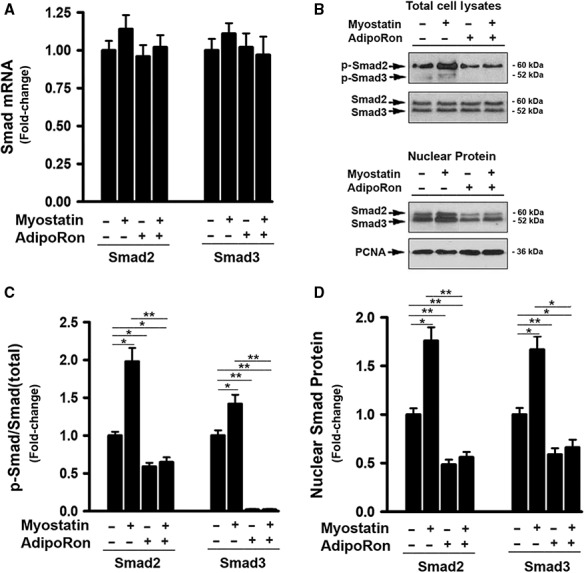
AdipoRon prevented MYOSTATIN‐induced activation of Smad2/3 signaling. (A) Cells were treated with either vehicle or myostatin (100 ng/mL), or AdipoRon (20 *μ*mol/L) for 2d, total RNA was isolated and subjected to Rt‐PCR analysis. (B) Cells were treated with either vehicle or myostatin (100 ng/mL), or AdipoRon (20 *μ*mol/L) for 3d. Total cell lysates (upper panel) and Nuclear protein (low panel) were prepared and subjected to Western blotting. (C) Blots in B (upper panel) were quantified by scanning densitometry and the ratio of phosphor‐Smad2 and 3 to endogenous Smad2 and 3, respectively, was calculated and is shown as fold of change. (D) Blots in B (low panel) were quantified by scanning densitometry and the ratio of nuclear Smad2 and 3 protein to PCNA (loading control) was calculated and is shown as fold of change.

Mstn acts locally or systemically via binding to the activin type II A and B (ActRIIA/B) receptors that activate ALK5 leading to phosphorylation of Smad2 and Smad3, which are then translocated to the nucleus to regulate gene expression (Thomas et al. 2000; Rebbapragada et al. 2003; Ricaud et al. 2003; Zhu et al. 2004; Han et al. 2013). The effect of AdipoRon on Smad2/3 activation was therefore examined. While AdipoRon treatment had no effect on endogenous Smad2/3 mRNA and protein expression (Fig. 5A and B), it significantly inhibited Mstn‐induced Smad2/3 phosphorylation in total cell lysates. In addition, AdipoRon reduced nuclear accumulation of Smad2/3 (Fig. 5B and D).

## Discussion

In this study, we tested whether Mstn directly alters insulin action and fatty acid synthesis, accumulation, and signaling pathways governing fatty acid synthesis in a hepatocyte line. We also tested the ability of AdipoRon to abrogate Mstn‐induced alterations in those cells. Our results revealed that Mstn reduced insulin action as reflected by lower IRS‐1 and IRS‐2 mRNA and protein expression and diminished insulin‐induced activating phosphorylation of IRS‐1 in mouse FL83B hepatocytes. These observations are consistent with the reports from other laboratories that Mstn inhibits hepatocellular insulin effect on glucose uptake (Wilkes et al. 2009; Watts et al. 2014). Mstn also elevated hepatocellular triglyceride levels which was associated with lower levels of inhibitory phosphorylation of ACC, increased FAS and perilipin2 expression, as well as suppression of PPAR*α* levels and DNA‐binding capacity. Regardless of the underlying mechanisms, the findings indicate that Mstn induces multiple perturbations of hepatocellular fatty acid homeostasis that result in accumulation of intracellular lipids. These findings are consistent with observations that a myostatin inhibitory peptide reduced hepato‐steatosis and expression of genes for lipid and cholesterol synthesis, including FAS and HMG‐Co‐reductase (Guo et al. 2013). Given the potential deleterious effects of accumulation of fats in hepatocytes observed herein, our findings suggest one possible mechanism for recent links between serum Mstn levels and liver diseases (Garcia et al. 2010; Nishikawa et al. 2017).

Adiponectin has been found to have beneficial actions on liver including insulin sensitizing effects and improved lipid homeostasis in a model of diet‐induced obesity (Gamberi et al. 2018). Moreover, over‐expression of AdipoR2 in mice improved NASH whereas inhibition of this receptor increases hepatocellular lipid peroxidation (Tomita et al. 2008). The data presented herein indicate that Mstn has antagonistic effects on adiponectin action. Specifically, Mstn downregulated AdipoR1, AMPK activation and PGC1*α*, as well as AdipoR2 and PPAR*α* expression and DNA‐binding capacity. Therefore, when interpreting these findings, one must consider Mstn as candidate responsible for the downregulation of adiponectin receptors in liver of patients with non‐alcoholic steatosis (Kaser et al. 2005). Mechanisms for these effects of Mstn on expression of AdipoR1/2, PPAAR*α* and PGC1*α*, and on signaling downstream of the receptors, are unclear but, as might be expected, were associated with activating phosphorylation of Smad2/3 and their nuclear migration. As noted above, Smad2/3 are the final effectors of Mstn action and would be expected to regulate the expression of a diverse array of genes. AdipoR1/2, PPAR*α*, and PGC1*α* appear to be among the genes regulated by these transcription factors although it remains uncertain whether such regulation is direct or indirect via modulation of other transcription factors, co‐regulators or effectors.

Of note, AdipoRon was capable of, at least in part**,** antagonizing Mstn‐induced alterations in hepatic gene expression, insulin signaling and fatty acid homeostasis, as well as suppression of signaling via AdipoR1 and R2. Importantly, the activation of Smad2/3, the ultimate effectors of Mstn action, was prevented by AdiopRon, providing one logical explanation as to how AdipoRon counteracts Mstn actions. However, the specific molecular mechanisms by which AdipoRon inhibits Mstn action in hepatocytes remain uncertain. Further studies are needed to determine whether AdipoRon affects downstream targets of Smad, such as FOXO1. Even so, the data provide evidence of multiple points of crosstalk between adiponectin and Mstn with regard to their respective effects on insulin action and homeostasis of fat metabolism in hepatocytes.

One of the more intriguing findings in the current study was the suppression of PPAR*α* expression and DNA binding capacity by Mstn. The effects of Mstn on PPAR*α* expression in adipose tissue deposits and skeletal muscle have also been reported. Inhibition of Mstn in transgenic mice overexpressing a myostatin pro‐peptide increased PPAR*α* expression in epididymal but not mesenteric fat (Suzuki et al. 2008). Conversely, Mstn knockouts or overexpression of Mstn pro‐peptide decreased PPAR*α* and PPAR*γ* expression in skeletal muscle (Mouisel et al. 2014) associated with conversion to a more glycolytic profile with lower endurance. One teleologic explanation for these differences between effects of Mstn on liver and epididymal fat as compared to muscle may be that Mstn knockouts reduce hepatic output of fats decreasing their availability to muscle resulting in lowered need for ß‐oxidation of fats, reduced expression of PPAR*α*/*γ* and diminished expression of enzymes for ß‐oxidation in muscle. While this proposal may not be correct, it is clear from the differential effects of Mstn on PPAR*α* expression in muscle as opposed to epididymal fat, mesenteric fat and liver, that there are contextual signals other than Mstn that regulate PPAR*α* expression and are dependent on tissue type.

The present study provides evidence regarding possible cellular and molecular mechanisms for the respective counter‐regulatory influences of Mstn and adiponectin on hepatocellular responses to cellular metabolism of fats and insulin action. In particular, the findings from the present study highlight the diverse effects of activation of Smad2/3 as a result of Mstn signaling on cellular metabolism, including not only Akt action, but also signaling via AMPK, PGC1*α*, and PPAR*α*. Given the growing evidence linking these regulatory factors to liver energy homeostasis and the progression of NASH, the findings highlight the importance of the potential humoral crosstalk between fat tissue, muscle and liver via production and release of Mstn and adiponectin and the opposing actions of these regulatory factors.

## Conflict of Interest

None declared.

## Supporting information




**Figure S1.** Effect of myostatin on hepatocyte proliferation.

**Figure S2.** AdipoRon prevented myostatin‐induced inhibition in Glut1 mRNA expression.
