# Supplementary figures and images for: AdipoRon prevents myostatin‐induced upregulation of fatty acid synthesis and downregulation of insulin activity in a mouse hepatocyte line
Source: Physiol Rep. 2019 Jun 27;7(13):e14152. doi: 10.14814/phy2.14152 (PMC6597868; doi:10.14814/phy2.14152)

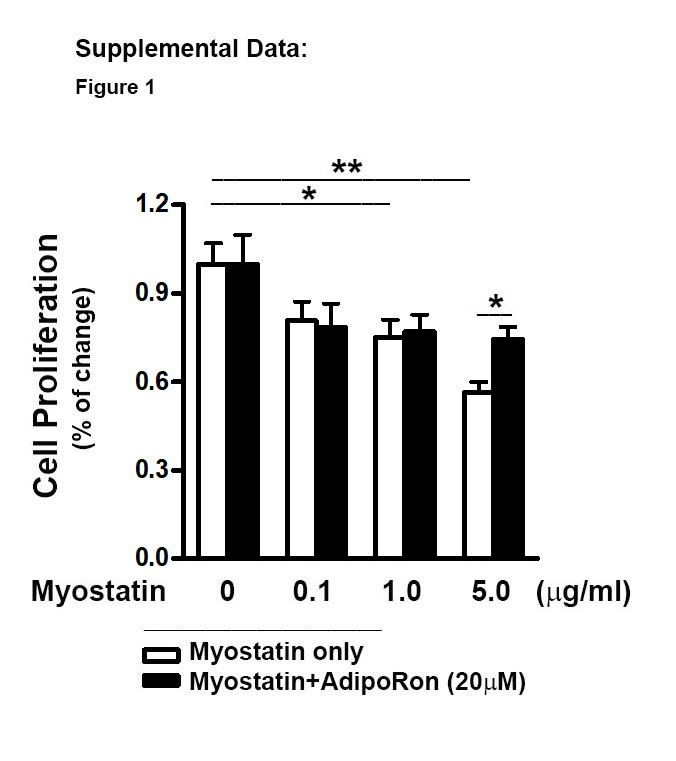

Supplement: Supplementary file 1 — Figure S1. Effect of myostatin on hepatocyte proliferation. [file PHY2-7-e14152-s001.jpg]

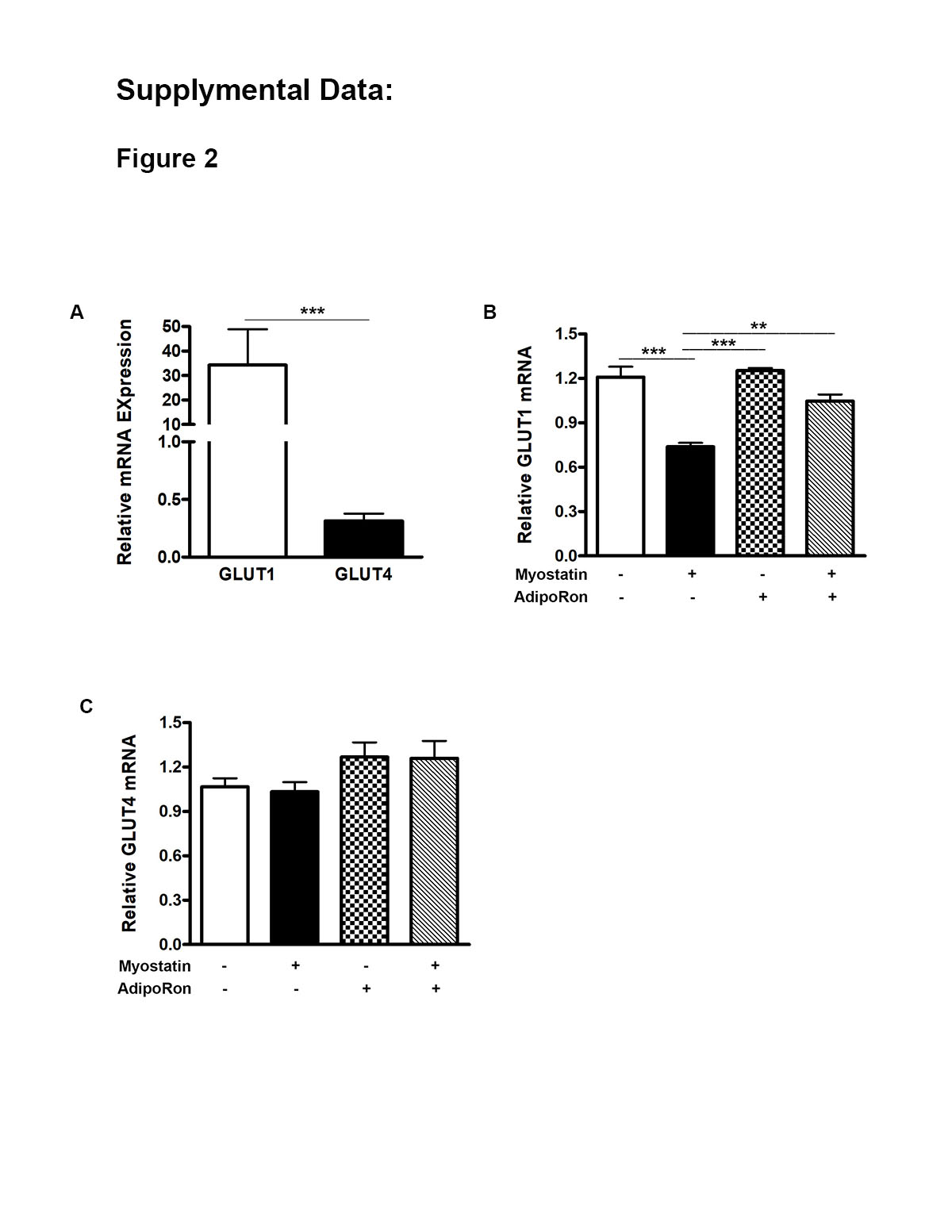

Supplement: Supplementary file 2 — Figure S2. AdipoRon prevented myostatin‐induced inhibition in Glut1 mRNA expression. [file PHY2-7-e14152-s002.jpg]
