# Supplementary material for: AdipoRon prevents myostatin‐induced upregulation of fatty acid synthesis and downregulation of insulin activity in a mouse hepatocyte line
Source: Physiol Rep. 2019 Jun 27;7(13):e14152. doi: 10.14814/phy2.14152 (PMC6597868; doi:10.14814/phy2.14152)
Supplement: Supplementary file 3 [file PHY2-7-e14152-s003.docx]

**Supplemental Data**

**Figure Legends:**

**Figure 1**. *Effect of Myostatin on Hepatocyte Proliferation*. FL83B cells were cultured at a density of 2.5 x 10^3^ cells per well in a flat-bottomed 96-well plate with F-12K medium and treated with various doses of myostatin, as indicated, in the presence or absence of AdipoRon (20μM) for 2d. Cell proliferation was assayed using the MTT Cell Proliferation Assay kit from ATCC (Cat No. 30-1010K). The assay was performed in triplicate and culture medium was used as blank control. * *p* < 0.05; ** *p* < 0.01.

**Figure 2.** *AdipoRon Prevented Myostatin-Induced Inhibition in Glut1 mRNA Expression*.

FL83B cells were treated with myostatin (100 ng/ml) and AdipoRon (20 μM), alone or in combination, for 2d. Total RNA was isolated and subjected to real-time PCR assay. * *p* < 0.05; ** *p* < 0.01; *** *p* < 0.001.
